# Editorial

**Published:** 2017

**Authors:** VL Purcarea

**Affiliations:** * "Carol Davila" University of Medicine and Pharmacy, Bucharest, Romania

**The European Federation of NeuroRehabilitation Societies (EFNRS)** is the most representative European organization dedicated to neurorehabilitation, an international non-profit organization centered on research, education, intellectual and scientific exchanges, advocacy, and philanthropic activities in the medical field of neurorehabilitation and of the related expertise branches. 

With its permanent headquarters in Vienna, **EFNRS** includes all the neurorehabilitation societies in the European Union countries, to which experts and scientific researchers in the whole world, dedicated to this special and important field, are added.

According to the statutory provisions, the election of the new president of the European Federation of NeuroRehabilitation Societies, for a two years mandate, took place at the end of last month, during the Fourth Edition of the European Congress of NeuroRehabilitation (ECNR), in Switzerland, Lausanne.

As a new recognition of the continuity of performances of the Romanian doctors and of the Romanian School of Medicine in the world medical field, Romanian Neurology received another confirmation of its incontestable value through the election of Prof. Dafin Mureşanu, MD, PhD, in this honorary position.

**Fig. 1:**
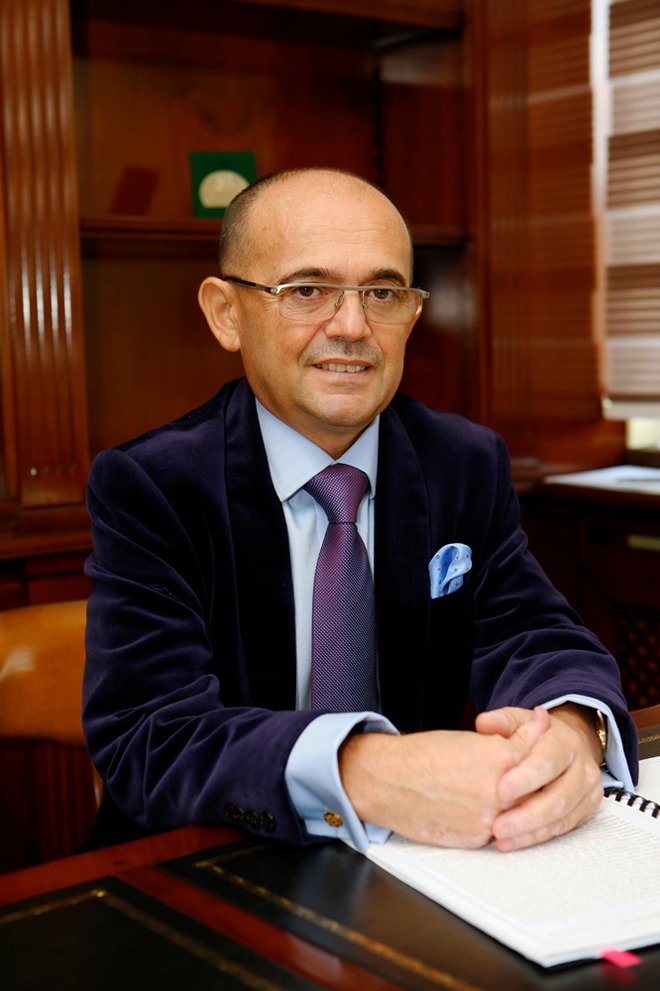
Professor Dafin Fior Mureşanu, MD, PhD

This event is presented in a news bulletin of the European Federation of NeuroRehabilitation Societies as ***“A new and prestigious recognition of value and professional completion of the Romanian School of NeuroRehabilitation at an European level, confirmed by the vote offered to Prof. Dafin Fior Mureşanu, MD, PhD, of leading, as president, the European Federation of NeuroRehabilitation Societies (EFNRS), the most representative European organization dedicated to neurorehabilitation”***.

Before his election as president, Prof. Dafin Mureşanu, MD, PhD, was vice-president of EFNR and, for almost ten years, he coordinated the whole package of educational programs at a European level, also contributing to the development strategy of the modules in the future educational program, dedicated to obtaining the competency in neurorehabilitation.

Prof. Dafin Mureşanu, MD, PhD, is currently the director of the Department of Neurosciences in “Iuliu Haţieganu” University of Medicine and Pharmacy, Cluj Napoca, member of the Academy of Medical Sciences in Romania, co-president of the Scientific Panel of Neurorehabilitation of the European Academy of Neurology, president of the Society for the Study of Neuroprotection and Neuroplasticity, and, until May this year, he was president of the Romanian Society of Neurology.

“During the last 10 years I have been working so hard for this European Federation, in which all the neurorehabilitation societies in each European state are included. I held the position of vice-president, dealing with aspects and projects regarding education and communication and, during this biannual congress that took part in Lausanne, I was, to my surprise, proposed and elected president of the European Federation, despite some dislikes from the Nordic countries, let’s say, structural, towards Romania, I did not receive any negative vote. This represents a new beginning, horizon, and recognition for the neurorehabilitation in Romania. No matter what, I consider that I have done many good things and I have opened many paths in this field. However, I believe that we will be looked at differently and this has already started happening. In this new position, I intend to promote an educational program at the level of a European MBA program, which should be able to offer a position and sketch the framework of this subspecialty, which we would like to turn into specialty. Moreover, my intention is that, together, we will place Romania better on the world map. The vote received from the EFNRS members honors me and, at the same time, makes me fulfill my promises and duties. My election in such an important position is the result of a decade of team work both at a European and at an international level”, declared Prof. **Dafin Mureşanu**, MD, PhD, with modesty.

*Distinguished Professor Dafin Mureşanu considers that Romania has a high potential for top research, for the investigation and study of new directions, in the benefit of the patients, and can be and must be a good partner for the most important groups of international research.*

„***Through the same effort sustained by the whole team at the EFNRS level we will continue the project of the integrated approach of neurorehabilitation in all its dimensions, motor, cognitive, and emotional. This desideratum cannot be reached without a proportional contribution of all the specialties involved, eventually in the reconstruction of the human being in all its dimensions after the great existential impact, the suffering due to the disease***”, noted the new president of the European Federation of NeuroRehabilitation Societies, at the beginning of his mandate.

**Executive Editor** **Professor Eng. Victor Lorin Purcarea, PhD.**

